# The RNA-binding protein ELAVL1 promotes Beclin1-mediated cellular autophagy and thus endometrial cancer development by affecting LncRNA-neat stability

**DOI:** 10.1080/15384047.2025.2469927

**Published:** 2025-02-28

**Authors:** Yanlu Luo, Xueyan Zhong, Xinzhao Sun, Jiangtao Fan

**Affiliations:** Department of Gynecology, First Affiliated Hospital of Guangxi Medical University, Nanning, Guangxi Zhuang Autonomous Region, P.R. China

**Keywords:** ELAVL1, endometrial cancer, autophagy, LncRNA-NEAT

## Abstract

Our study aims to investigate the roles of embryonic lethal abnormal vision-like 1 (ELAVL1) and long non-coding RNA (LncRNA) NEAT1 in endometrial cancer (EC), focusing on their underlying molecular mechanisms.We obtained EC cell lines (HEC-1A, Ishikawa, RL95–2, HEC-1B, and AN3CA) from ATCC. We used siRNAs (si-ELAVL1#1 and si-ELAVL1#2) and overexpression RNAs (OE ELAVL1 and OE-NEAT1) for knockdown or overexpression of ELAVL1 and LncRNA NEAT1. We also employed 3-MA (5mM) or rapamycin (100µM) to inhibit or promote autophagy. Moreover, we conducted RNA immunoprecipitation (RIP) assays to confirm the interaction between LncRNA NEAT1 and ELAVL1. Cell Counting Kit-8 (CCK-8) and transwell assays were utilized to assess cell proliferation and migration. Additionally, we measured the expression of ELAVL1 and Beclin1 through Western blotting and RT-qPCR.ELAVL1 was found to be highly expressed in EC. Furthermore, ELAVL1 promoted the proliferation, invasion, and migration of EC cells through the regulation of Beclin1-related pathways. RIP assays revealed a direct interaction between LncRNA NEAT1 and ELAVL1, with ELAVL1 stabilizing LncRNA NEAT1 mRNA in EC cells. Additionally, we observed that ELAVL1 influenced EC cell proliferation, invasion, and migration through the regulation of LncRNA NEAT1-mediated regulation of Beclin1 expression. Moreover, in an animal study, we determined that ELAVL1 influenced endometrial cancer tumor growth through its interaction with LncRNA NEAT1, which mediated Beclin1 expression in vivo.In summary, our study showed that ELAVL1 regulated the malignant behavior of endometrial cancer cells through the modulation of LncRNA NEAT1-mediated regulation of Beclin1 expression.

## Introduction

Endometrial cancer (EC) is one of the most common gynecological tumors worldwide, ranking second only to breast cancer.^1,2^ It is estimated that in 2018, there were over 380,000 newly diagnosed cases of EC worldwide, with approximately 90,000 deaths attributed to this disease.^3^ The most recent epidemiological research indicates a significant annual increase of 0.69% in the age-standardized incidence rate of EC from 1990 to 2019.^4,5^ Risk factors associated with EC incidence include age, obesity, sedentary lifestyle, diabetes, and hormone usage.^6–8^ Despite the fact that the 5-year survival rate for most EC cases exceeds 90%,^9^ emphasizing the need for a deeper understanding of its molecular mechanisms.

Cellular autophagy, the process of self-degradation and recycling, has been implicated in cancer progression.^10,11^ In the context of EC, the regulation of cellular autophagy can impact cell proliferation, apoptosis, and immune evasion.^12–14^ Clinically, receiver operating characteristic (ROC) analysis has shown that mRNA levels of autophagy-related genes can accurately predict prognosis in EC patients.^15^ Human antigen R (HuR), encoded by the embryonic lethal abnormal vision-like 1 (ELAVL1) protein, a member of the EL AV family, is an RNA-binding protein that stabilizes mRNA and promotes gene expression.^16^ ELAVL1 has been reported to play a key role in many cellular processes, including angiogenesis, apoptosis and inflammation.^17,18^ Stabilization of ELAVL1 protein promotes proliferation, metastasis, and chemotherapy resistance in breast cancer.^19^ ELAVL1 directly binds to Beclin-1 mRNA and enhances its stability, whereas silencing ELAVL1 can inhibit autophagy.^20^ Moreover, Studies have indicated that in cancer cells, activated ELAVL1 may promote invasion and metastasis by regulating autophagy, thereby driving cancer progression.^21,22^ TCGA database analysis has revealed an upregulation of ELAVL1 expression in EC. Moreover, a recent study confirmed that targeting ELAVL1-related signaling pathways can enhance the malignant behavior of EC cells.^23^ However, there is a lack of research investigating the mechanisms of ELAVL1 in EC and its relationship with cellular autophagy.

In our previous research, we discovered that overexpression of LncRNA NEAT1 accelerates tumor growth in EC in *vivo*.^24^ Now, we aim to investigate the RNA-binding protein(s) associated with LncRNA NEAT1 to elucidate its molecular mechanisms in EC development. Through bioinformatics analysis using the RBPDB database, we identified multiple binding sites between LncRNA NEAT1 and ELAVL1. We hypothesize that ELAVL1 may regulate the mRNA stability of LncRNA NEAT1 and influence the progression of EC through cellular autophagy. Our study aims to explore the roles of ELAVL1 and LncRNA NEAT1 in EC, as well as their potential molecular mechanisms.

## Methods

### Cell treatment

The EC cell lines (HEC-1A, Ishikawa, RL95–2, HEC-1B and AN3CA) were obtained from ATCC (Manassas, Virginia). The cell line was tested for mycoplasma contamination and identified by short tandem repeat (STR) analysis. At 37°C and 5% CO_2_, the RPMI-1640 medium (Thermo Fisher Scientific, Inc., MA, USA), which contained 10% Gibco® fetal bovine serum (FBS, Gibco, MD, USA) and 100 µg/mL penicillin-streptomycin, was used for cells culture. To inhibit or promote autophagy, cells were treated with 3-MA (5 mm) or rapamycin (100 µM). In addition, for detecting the stability of the RNA of LncRNA NEAT1, after treating the cells with actinomycinone cycloheximide (CHX, 20 μg/mL), the mRNA level of LncRNA NEAT1 was detected in each group of cells by RT-qPCR at 0/2/4/6/8 h of treatment, respectively.

### Cell transfection

The different siRNAs (si-ELAVL1#1 and si-ELAVL1#2), OE RNAs (OE-ELAVL1 and OE-NEAT1) and negative controls (si-NC and OE-NC) were procured from GeneChem Corp for use in cell transfection experiments. The sequences of siRNAs were shown in Table 1. The OE-ELAVL1 and OE-NEAT1 (Myc-DDK-tagged)-human cDNA clones were provided without sequence information.

**Table 1.** The sequences of all constructs.

Name	Sequence
si-ELAVL1#1	GAACGAAUUUGAUCGUCAATT
si-ELAVL1#2	AAGAGGCAAUUACCAGUUUCA

Following the protocol provided by the manufacturer, cells were transfected with 5 nM of the above si-RNAs, OE-RNAs or NCs following the manufacturer’s instructions. Transfection of the siRNAs and negative control into the EC cells were performed using Lipofectamine 3000 (Invitrogen, California, USA) reagent. We transfected the cells in serum-free Opti-MEM medium and measured the transfection efficiency via RT-qPCR 48 hours after transfection.

### RNA immunoprecipitation (RIP) assay

We conducted RIP assays by employing an EZ-Magna RIP™ RNA-Binding Protein Immunoprecipitation Kit (Millipore, Billerica, MA, USA) by the manufacturer’s guidelines. We lysed cells at approximately 90% confluence using a complete RIP lysis buffer that contained an RNase inhibitor (Millipore) and protease inhibitor. Then, we incubated 100 μl of whole cell extract with RIP buffer that contained magnetic beads conjugated to specific antibodies. The negative control consisted of a normal mouse anti-IgG antibody (Cell Signaling Technology, USA), whereas the positive control was an anti-SNRNP70 antibody (Millipore, USA).

### Cell counting kit-8 (CCK-8)

Cells were seeded into 96-well plates at a density of 5 × 10^3^ cells per well. After incubating the cells with CCK-8 reagent (10 μL, Sangon) for 2 hours, and the absorbance at 450 nm was determined on a microplate reader (Thermo Fisher Scientific, MA, USA).

### Transwell assay

For cell migration, the non-coated membrane was used to plate cell suspension containing 4 × 10^4^ cells/ml in the upper chamber (24-well insert; 8 mm; BD Biosciences). Then, 2 × 10^5^ cells were plated in the top compartment with a membrane coated with Matrigel for invasion experiments (24-well insert; 8 mm; BD Biosciences). The samples were incubated for 24 h, followed by staining of 0.1% crystal violet. Samples were then photographed under an optical microscope.

### Immunofluorescence staining (IFC)

Cells were fixed, permeabilized, and incubated with anti-Beclin1 antibody (1:100, Abcam) at 4°C overnight. Subsequently, cells were incubated with the appropriate secondary antibodies at room temperature for 1 hour. DAPI was used for cytoplasmic staining.

### RT-qPCR

RT-qPCR was used to measure the expression of ELAVL1, LncRNA NEAT1, and beclin1. Total RNA was extracted from cells using the RNAiso Plus reagent kit (Takara, Japan). The RNA was then converted to cDNA using the PrimeScript™ One Step RT-qPCR kit (Takara Biotechnology Co., Ltd., Dalian, China). Subsequently, qPCR analysis was performed using the SYBR Premix ExTaq (TaKaRa, Japan) on an ABI PRISM7300 Sequence Detection System (Applied Biosystems). Primer sequences were: ELAVL1 Forward 5’-GGGTGACATCGGGAGAACG-3’, Reverse 5’-CTGAACAGGCTTCGTAACTCAT-3’; LncRNA NEAT1 Forward 5’-GTACGCGGGCAGACTAACAC-3’, Reverse 5’-TGCGTCTAGACACCACAACC-3’; Beclin1 Forward 5’-CTGGTAGAAGATAAAACCCGGTG-3’, Reverse 5’-AGGTAGAGCGTGGACTATCCG-3’; GAPDH Forward 5′-CACCCACTCCTCCACCTTTG-3′, Reverse 5′-CCACCACCCTGTTGCTGTAG-3′. GAPDH served as an internal control. mRNA expression was calculated using the 2-^ΔΔ^Ct method.

### Western blot

Cells were lysed using RIPA lysis buffer (P0013D, Biyuntian, Shanghai, China) to extract total protein, which was quantified using the BCA method. Protein samples and standards were loaded onto 10% SDS-PAGE gels, which were separated and transferred onto polyvinylidene fluoride (PVDF) membranes. After blocking with 5% BSA for 1 hour, membranes were incubated overnight at 4°C with the appropriate primary antibody. The membranes were then incubated at 37°C with goat anti-rabbit IgG H&L secondary antibody (ab96899, 1/1000). The primary antibodies included ELAVL1 antibody (ab92310, 1/1000, Abcam), beclin1 antibody (ab207612, 1/2000, Abcam), LC3B (ab222776, 1/200, Abcam), p62 (ab109012, 1/10000, Abcam) and GAPDH (ab9485, 1/2500, Abcam) was served as an internal control. After incubation, protein bands were visualized using an X-ray imaging system. The membranes were developed and imaged using a chemiluminescence imaging system. The gray values of the strips were analyzed using Image J 6.0 software (Rasband; NIH, USA), using GAPDH as an internal reference.

### Tumor forming model

A total of 36 BALB/c nude mice were used to establish subcutaneous tumor models by injecting HEC-1A cells or HEC-1A cells transfected with siRNAs. A cell suspension containing 2 × 10^4^ cells in 0.2 ml was injected into the back of each mouse. Tumor volume was determined by measuring the length (l) and width (w) and calculating the volume (V) using the formula: V = lw2/2. After 21 days, the mice were euthanized, and the tumor tissues were weighed. This animal study was approved by the Animal Ethics Committee.

### Immunohistochemistry (IHC)

Tumor tissues were fixed in 4% paraformaldehyde, dehydrated, embedded in paraffin, and sectioned into consecutive 4 μm slices. Deparaffinization was performed using routine methods. IHC staining was conducted according to the standard protocol of the avidin-biotin-peroxidase complex method using an IHC staining kit. Subsequently, tissue sections were incubated overnight at 4°C with the anti-Beclin1 antibody (1:100, Abcam). The tissues were then incubated with the appropriate secondary antibodies for 30 minutes at 37°C. Following this, the tissues were incubated with an HRP-conjugated working solution and stained with 3,3’-diaminobenzidine for 5–10 minutes. The staining time was adjusted under a microscope. After counterstaining with hematoxylin for 1 minute, the tissues were fixed with mounting medium, dried, and photographed. Five high-power fields were selected for observation and counting under a bright-field microscope.

### Statistical analysis

The data is presented as mean ± SD, and we utilized one-way ANOVA followed by a Tukey post hoc test for statistical comparisons. For significance, a P-value less than 0.05 was considered.

## Results

### High expression of ELAVL1 in endometrial cancer and its role in Beclin1-mediated pathways

**Figure 1.**
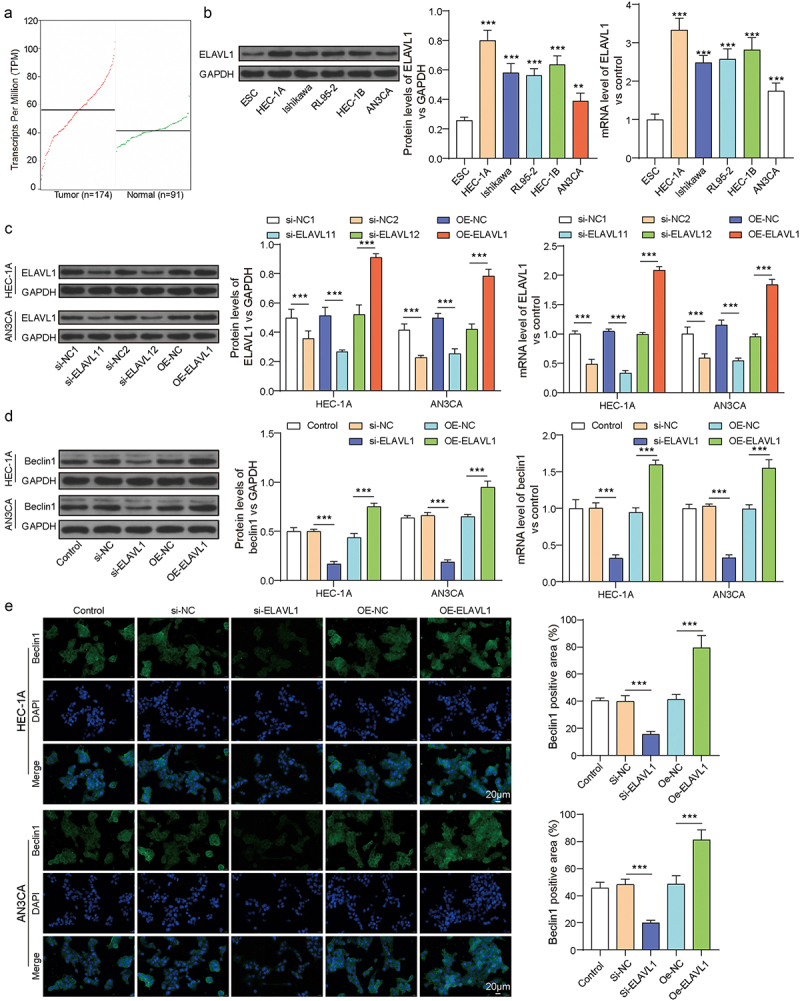
ELAVL1 was highly expressed in EC and promoted autophagy. (a) TCGA database shows high expression of ELAVL1 in EC. (b) The mRNA and protein levels of ELAVL1 in EC cell lines. (c) The mRNA and protein levels of ELAVL1 after EC cells transfection. (d) The mRNA and protein levels of beclin1 in EC cells of each group. (e) The expression of beclin1 was measured by IFC. Continuous data between two groups were compared using Student’s t-test, while comparisons among three or more groups were analyzed by one-way analysis of variance and Tukey’s post hoc test. **p<.01 compared with the corresponding group. Each experiment is repeated three times.

We first queried the TCGA database for the expression of ELAVL1 in endometrial cancer. The results in Figure 1a show that ELAVL1 was highly expressed in endometrial cancer according to the TCGA database. RT-qPCR and Western blotting results similarly demonstrated a significant enhancement of ELAVL1 expression in endometrial cancer cell lines (HEC-1A, Ishikawa, RL95–2, HEC-1B, and AN3CA) compared to ESC cells (Figure 1b, *p* < .05). In the cell lines with the highest (HEC-1A) and lowest (AN3CA) ELAVL1 expression, ELAVL1 expression was successfully knocked down using si-ELAVL1 and overexpressed using OE-ELAVL1 (Figure 1c, *p* < .05). Subsequently, we detected the expression of the autophagy gene beclin1 in HEC-1A and AN3CA cell lines. RT-qPCR, Western blotting and IFC results showed that silencing ELAVL1 reduced beclin1 mRNA and protein levels, whereas overexpressing ELAVL1 significantly increased beclin1 mRNA and protein levels (Figure 1d–e, *p* < .05). Additionally, silencing ELAVL1 significantly reduced LC3B protein expression while increasing p62 levels in HEC-1A cell line. Conversely, overexpression of ELAVL1 produced the opposite effects, further supporting its role in regulating autophagy (Supplementary Figure S1). This indicated that high ELAVL1 expression in endometrial cancer might facilitate cancer progression through its regulation of Beclin1.

### ELAVL1 promoted the proliferation, invasion, and migration of EC cells through the regulation of Beclin1-related pathways

**Figure 2.**
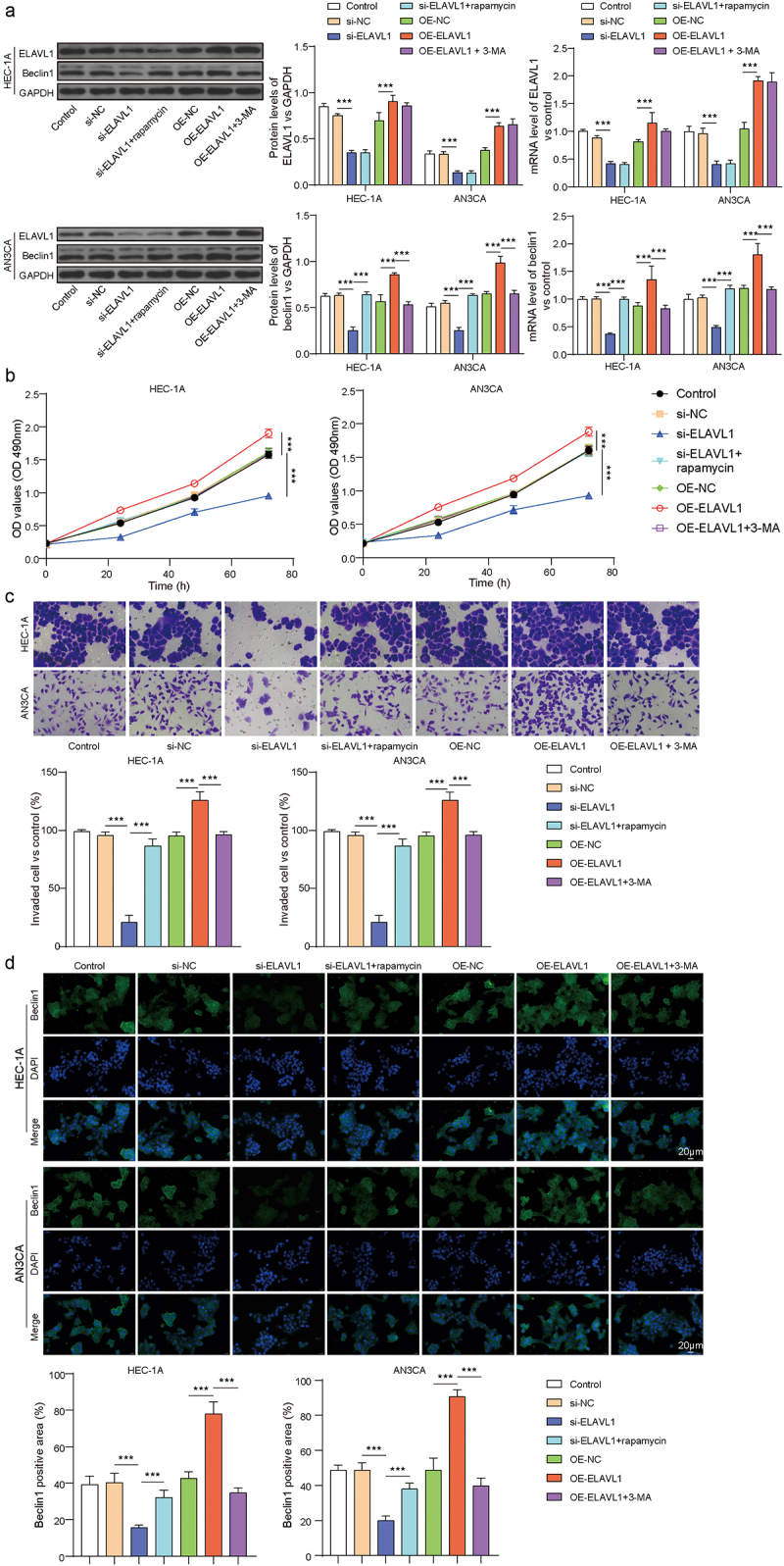
ELAVL1 promoted EC cell proliferation, invasion, and migration by regulating autophagy. (a) The mRNA and protein levels of ELAVL1 and beclin1 in each group. The EC cell viability and invasion in each group were measured by CCK-8 (b) and Transwell (c). The continuous data were compared using Student’s t test between two groups. (d) The expression of beclin1 was measured by IFC. ANOVA followed by Tukey’s post hoc test was used for comparison among three or more groups. **p<.01 compared with the corresponding group. Each experiment is repeated three times.

In HEC-1A and AN3CA cells, we used si-ELAVL1 or OE-ELAVL1 to silence or overexpress ELAVL1 and used 3-MA or rapamycin to inhibit or enhance cell autophagy. After detecting the expression of ELAVL1 and beclin1 in each group of cells using RT-qPCR and Western blotting, we found that, compared to si-NC, the si-ELAVL1 group showed a significant decrease in ELAVL1 and beclin1 mRNA and protein expression, which significantly recovered after treatment with rapamycin (Figure 2a–b, *p* < .05). Silencing ELAVL1 inhibited cell proliferation and migration, as shown by CCK-8 and transwell results (Figure 2b–c, *p* < .05). Additionally, rapamycin promoted autophagy and reversed the inhibitory effect on cell proliferation and migration, resulting in a significant recovery in these processes (*p* < .05). Furthermore, overexpressing ELAVL1 promoted cell proliferation and migration, and inhibiting autophagy with 3-MA significantly suppressed the proliferation and migration of EC cells (*p* < .05). We also detected the expression beclin1 using IFC, which showed similar results to the Western blotting (Figure 2e). Additionally, rapamycin treatment increased LC3B protein levels, whereas 3-MA suppressed them in the HEC-1A cell line, with p62 showing the opposite trend (Supplementary Figure S1). These findings indicate that ELAVL1 promoted the proliferation, invasion, and migration of EC cells through the regulation of Beclin1-related pathways.

### ELAVL1 stabilized LncRNA NEAT1 mRNA in EC cells

**Figure 3.**
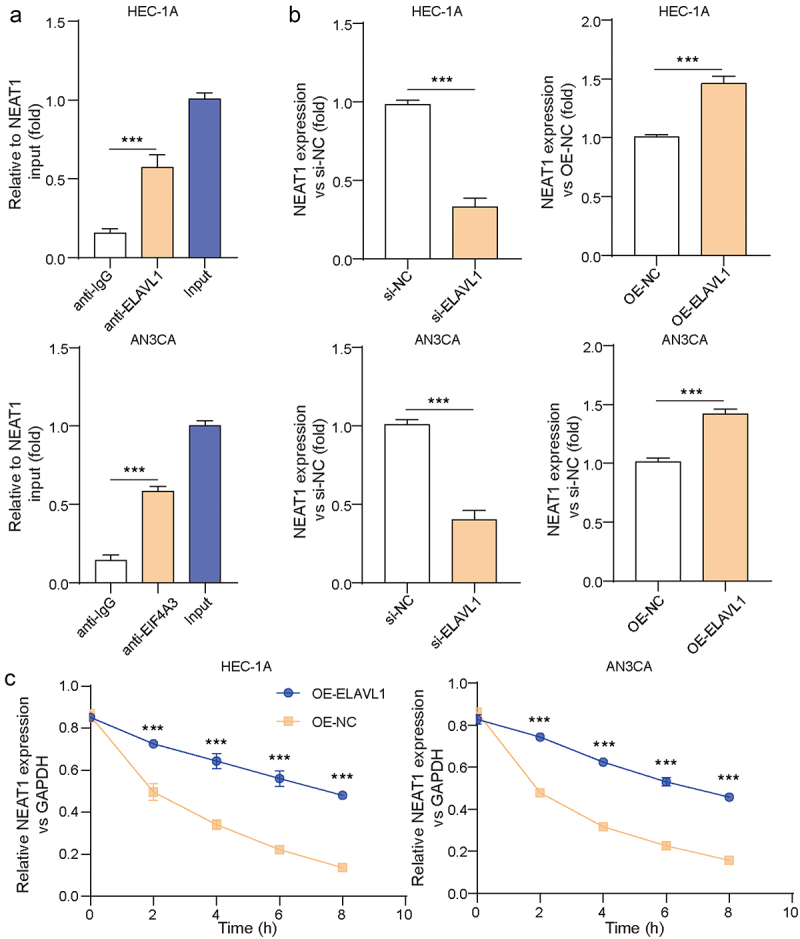
ELAVL1 binded to LncRNA NEAT1 and stabilized NEAT1 mRNA in EC cells. (a) RIP assay was performed to verify the binding relationship between ELAVL1 and LncRNA NEAT1. (b) The mRNA levels of LncRNA NEAT1 were measured by rt-qPCR. (c) Validation of ELAVL1 stable LncRNA NEAT1 mRNA expression by actinomycin D treatment. The continuous data were compared using Student’s t test between two groups. ANOVA followed by Tukey’s post hoc test was used for comparison among three or more groups. **p<.01 compared with the corresponding group. Each experiment is repeated three times.

We then explored the relationship between ELAVL1 and lncRNA NEAT1 in EC cells. RIP results showed that in HEC-1A and AN3CA cells, the enrichment level of LncRNA NEAT1 was significantly higher in the ELAVL1 group compared to the IgG group (Figure 3a, *p* < .001), indicating a direct interaction between LncRNA NEAT1 and ELAVL1. RT-qPCR results showed that inhibiting ELAVL1 decreased LncRNA NEAT1 expression while overexpressing ELAVL1 enhanced LncRNA NEAT1 expression (Figure 3b, *p* < .001). Treating the cells with actinomycin D for different periods, we found that overexpressing ELAVL1 could stabilize LncRNA NEAT1 mRNA expression, while silencing of ELAVL1 decreased LncRNA NEAT1 RNA stability (Figure 3c). These findings confirmed that ELAVL1 could bind to LncRNA NEAT1 and stabilize NEAT1 mRNA in EC cells.

### ELAVL1 affected EC cell proliferation, invasion, and migration through the regulation of LncRNA NEAT1-mediated regulation of Beclin1 expression

**Figure 4.**
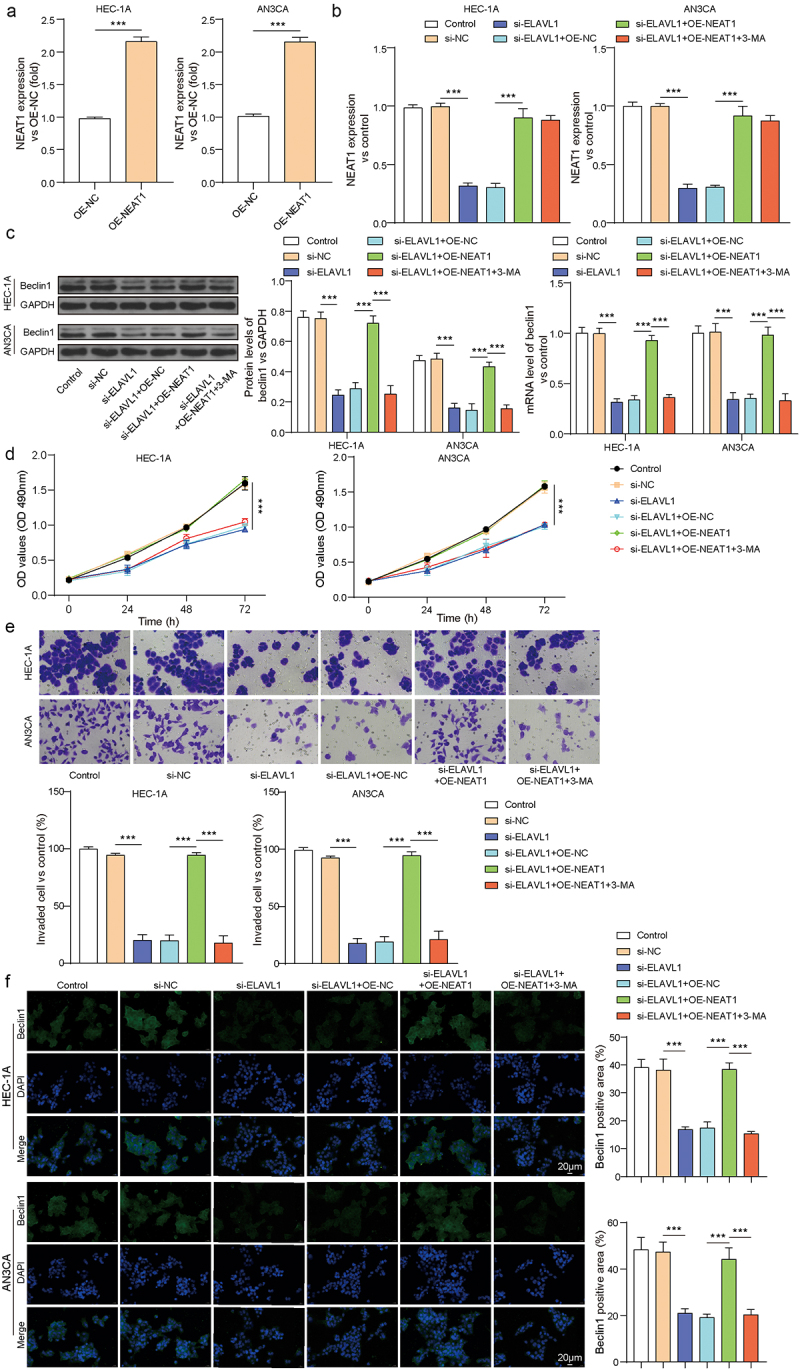
ELAVL1 affected EC cell proliferation, invasion, and migration by regulating NEAT1-mediated autophagy. (a) RT-qPCR detection of LncRNA NEAT1 transfection efficiency. (b) The mRNA levels of LncRNA NEAT1 were measured by rt-qPCR after cells transfection. (c) The protein and mRNA expression of beclin1 in each group. The EC cell viability and invasion in each group were measured by CCK-8 (d) and Transwell (e). The continuous data were compared using Student’s t test between two groups. (F) The expression of beclin1 was measured by IFC. ANOVA followed by Tukey’s post hoc test was used for comparison among three or more groups. **p<.01 compared with the corresponding group. Each experiment is repeated three times.

Furthermore, in HEC-1A and AN3CA cells, we used si-ELAVL1 to silence ELAVL1, OE-NEAT1 to overexpress LncRNA NEAT1, and 3-MA to inhibit cell autophagy to investigate the effect of ELAVL1 regulation of LncRNA NEAT1 on cell autophagy-mediated proliferation, invasion, and migration of EC cells. RT-qPCR results showed that OE-NEAT1 transfection significantly enhanced LncRNA NEAT1 mRNA expression (Figure 4a, *p* < .001). Silencing ELAVL1 reduced LncRNA NEAT1 expression, and overexpressing LncRNA NEAT1 reversed this effect (Figure 4b, *p* < .001), while inhibiting autophagy with 3-MA did not significantly change LncRNA NEAT1 mRNA expression when compared to the si-ELAVL1 + OE-NEAT1 group. Silencing ELAVL1 reduced the expression of the autophagy gene beclin1, while overexpressing OE-NEAT1 reversed this effect, resulting in a significant increase in beclin1 expression (Figure 4c–d, *p* < .001). Furthermore, overexpression of NEAT1 increased LC3B protein levels and reduced p62 levels, effects that were reversed by 3-MA in the HEC-1A cell line (Supplementary Figure S1).

In addition, CCK-8 and transwell assays detected the proliferation and invasion of EC cells. As shown in Figure 4e–f, silencing ELAVL1 inhibited EC cell proliferation and invasion, while overexpressing LncRNA NEAT1 promoted these processes (*p* < .001). This effect was reversed by inhibiting cell proliferation with 3-MA, as the si-ELAVL1 + OE-NEAT1 + 3-MA group showed significant inhibition of cell proliferation and invasion when compared to the si-ELAVL1 + OE-NEAT1 group (*p* < .001). These findings confirmed that ELAVL1 influences the proliferation, invasion, and migration of EC cells by regulating LncRNA NEAT1-mediated regulation of Beclin1 expression.

### ELAVL1 influences endometrial cancer tumor growth through NEAT1-dependent regulation of Beclin1 expression

**Figure 5.**
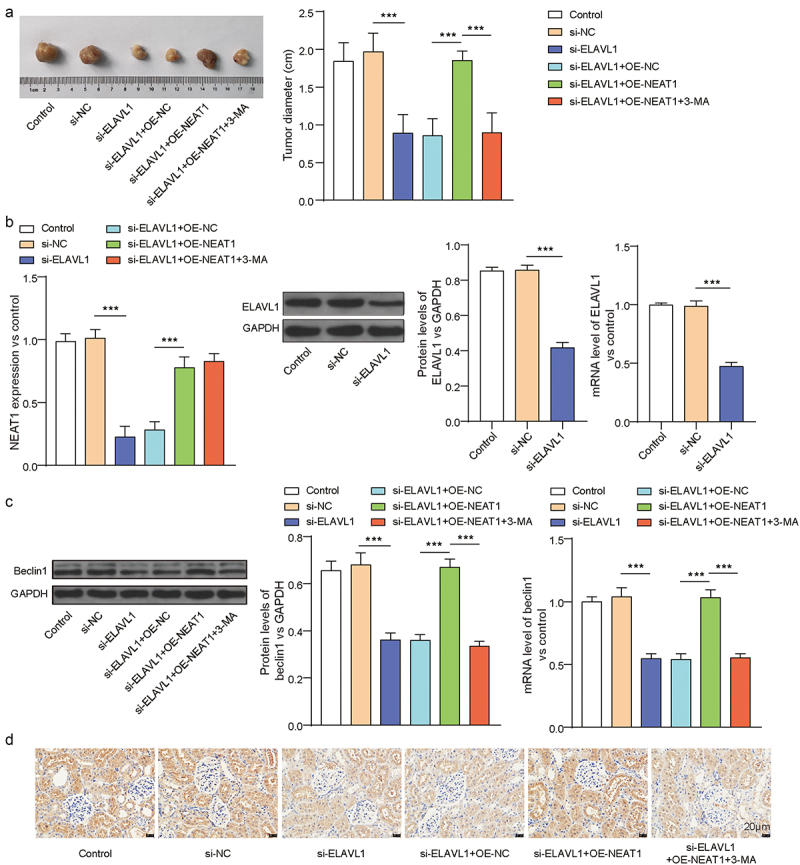
ELAVL1 affected tumor growth in EC by regulating NEAT1-mediated cellular autophagy. (a) Tumor volume in each group of mice. (b) The protein levels of ELAVL1 and mRNA levels of ELAVL1, LncRNA NEAT1 were measured by Western blotting and rt-qPCR. (c) The protein levels of beclin1 were measured by Western blotting. (d) The expression of beclin1 was measured by IHC. The continuous data were compared using Student’s t test between two groups. ANOVA followed by Tukey’s post hoc test was used for comparison among three or more groups. **p<.01 compared with the corresponding group. 6 mice per group.

Finally, we investigated the effect of ELAVL1 on EC tumor growth in vivo. As shown in Figure 5a compared to the si-NC group, the tumor volume of mice in the si-ELAVL1 group was significantly reduced (*p* < .001), which was reversed by overexpressing LncRNA NEAT1 (*p* < .001). Using RT-qPCR and Western blotting to detect ELAVL1, LncRNA NEAT1, and beclin1 expression in tumor tissues of each group of mice, we found that compared to the si-NC group, the tumor tissues of mice in the si-ELAVL1 group showed a significant decrease in ELAVL1 and LncRNA NEAT1 expression (Figure 5b *p* < .001), and inhibiting autophagy did not change the expression of ELAVL1 and LncRNA NEAT1. In addition, as expected, inhibiting ELAVL1 significantly reduced the expression of the autophagy gene beclin1, while overexpressing LncRNA NEAT1 reversed this effect, resulting in a significant increase in beclin1 expression (Figure 5c–d, *p* < .001). These data demonstrated that in vivo, ELAVL1 influenced endometrial cancer tumor growth through its interaction with LncRNA NEAT1, which mediated Beclin1 expression.

## Discussion

Despite the effectiveness of surgical treatment for early-stage breast cancer, the prognosis for stage III-IV EC patients remains poor, with low 5-year overall survival rates.^25^ These patients are at a higher risk of recurrence and metastasis, highlighting the critical need to understand the molecular mechanisms underlying EC progression and identify new therapeutic targets. In our study, we discovered that ELAVL1 plays a significant role in regulating the malignant behavior of endometrial cancer cells through the modulation of LncRNA NEAT1-mediated regulation of Beclin1 expression.

Autophagy, a process involved in self-degradation and recycling, has a dual role in tumor progression, either suppressing or promoting cancer development depending on the stage.^10,26^ In advanced cancer, autophagy has been shown to enhance the survival and growth of cancer cells, as well as confer resistance to chemotherapy drugs.^27,28^ Autophagy can modulate antigen presentation, immune cell function, and cytokine production, ultimately influencing tumor immune evasion.^29^ In our investigation, we examined the relationship between the autophagy factor beclin1 and the malignant behavior of EC cells. Our results demonstrated that increased expression of beclin1 was associated with enhanced malignant behavior of EC cells, including proliferation, invasion, and tumor growth, both in vitro and in vivo. This indicated that autophagy promoted disease progression in EC cells. Therefore, it is plausible to speculate that autophagy may also contribute to immune evasion in our model. Specifically, it has been reported that autophagy can regulate the expression of immune checkpoint molecules such as PD-L1, which can suppress the activity of cytotoxic T cells and facilitate immune escape.^30^ Given that ELAVL1 and LncRNA NEAT1 were found to regulate autophagy in our study, as its overexpression significantly increased the expression of beclin1, an autophagy-related gene, it is conceivable that this autophagy-mediated regulation could influence the expression of immune checkpoint molecules and impact immune evasion mechanisms in endometrial cancer. Previous studies by Li *et al*. demonstrated that LncRNA NEAT1 promotes autophagy in liver cancer cells by regulating the miR-204/ATG3 pathway.^31^ Wang *et al*. suggested that LncRNA NEAT1 promoted autophagy by regulating several autophagy-related genes, including beclin1.^32^ These findings were consistent with ours, additionally, Wang *et al*. identified 14 autophagy-related LncRNAs with significant prognostic value for endometrial cancer by Pearson correlation analysis of 1297 autophagy-related LncRNAs.^33^ These findings suggested that autophagy related LncRNAs may play a crucial role in the occurrence and development of endometrial cancer.

Furthermore, we investigated the RNA-binding protein of LncRNA NEAT1 and found that ELAVL1 interacts with LncRNA NEAT1 and stabilizes its mRNA in EC cells. ELAVL1 plays a crucial role in post-transcriptional gene regulation.^34^ HIF-1α, TNF, and other mRNAs have been shown to bind to ELAVL1, suggesting that ELAVL1 is involved in many processes, including apoptosis, inflammatory and oxidative stress.^35^ Previous studies have reported on the interaction between ELAVL1 and LncRNAs. For instance, silencing of LncRNA PARAIL enhances the function of ELAVL1, exacerbating inflammatory responses in inflammatory diseases.^36^ Upregulation of LncRNA RMRP enhances ELAVL1 expression, suppressing cell viability, promoting apoptosis, and exacerbating oxidative stress and inflammatory responses in acute kidney injury.^37^ In cancer, LncRNA SNHG7 influences the malignant behavior of nasopharyngeal carcinoma cells through the miRNA/ELAVL1 axis.^38^ Additionally, in ischemia-reperfusion injury, idiopathic pulmonary fibrosis, and hepatocellular carcinoma, ELAVL1 has been shown to participate in autophagy by regulating the expression of autophagy-related proteins.^20,39,40^ However, the relationship between ELAVL1 and autophagy in EC has not been explored. In this study, we discovered that ELAVL1 serves as a novel regulatory factor of autophagy in EC cells. Our findings demonstrated that ELAVL1 positively regulated the expression of LncRNA NEAT1, which, in turn, promoted autophagy by upregulating beclin1 expression, thereby exacerbating the malignant behavior of EC cells.

Although our findings shed light on the role of ELAVL1 and LncRNA NEAT1 in EC progression, there are still some limitations to our study. The regulation of ELAVL1 by other genes requires further investigation, and the impact of ELAVL1 and LncRNA NEAT1-mediated autophagy on apoptosis in EC cells was not experimentally explored. These aspects warrant future research to deepen our understanding of the molecular mechanisms underlying EC and identify potential therapeutic targets.

## Conclusions

In summary, our study showed that ELAVL1 regulated the malignant behavior of endometrial cancer cells through the modulation of LncRNA NEAT1-mediated regulation of Beclin1 expression. Our findings revealed the molecular mechanisms underlying the progression of endometrial cancer and highlighted potential therapeutic targets.

## Supplementary Material

Supplemental Material

FIGURE S1.jpg

## Data Availability

All data generated or analyzed during this study are included in this published article.
