# Supplementary material for: The RNA-binding protein ELAVL1 promotes Beclin1-mediated cellular autophagy and thus endometrial cancer development by affecting LncRNA-neat stability
Source: Cancer Biol Ther. 2025 Feb 28;26(1):2469927. doi: 10.1080/15384047.2025.2469927 (PMC11875488; doi:10.1080/15384047.2025.2469927)
Supplement: Supplemental Material [file KCBT_A_2469927_SM3890.docx]

**Supplementary Figure:**

**Figure S1.** Alteration of LC3B and p62 under different conditions in *in vitro* experiments in HEC-1A cell line. ***P<0.001.
