# Supplementary figures and images for: The RNA-binding protein ELAVL1 promotes Beclin1-mediated cellular autophagy and thus endometrial cancer development by affecting LncRNA-neat stability
Source: Cancer Biol Ther. 2025 Feb 28;26(1):2469927. doi: 10.1080/15384047.2025.2469927 (PMC11875488; doi:10.1080/15384047.2025.2469927)

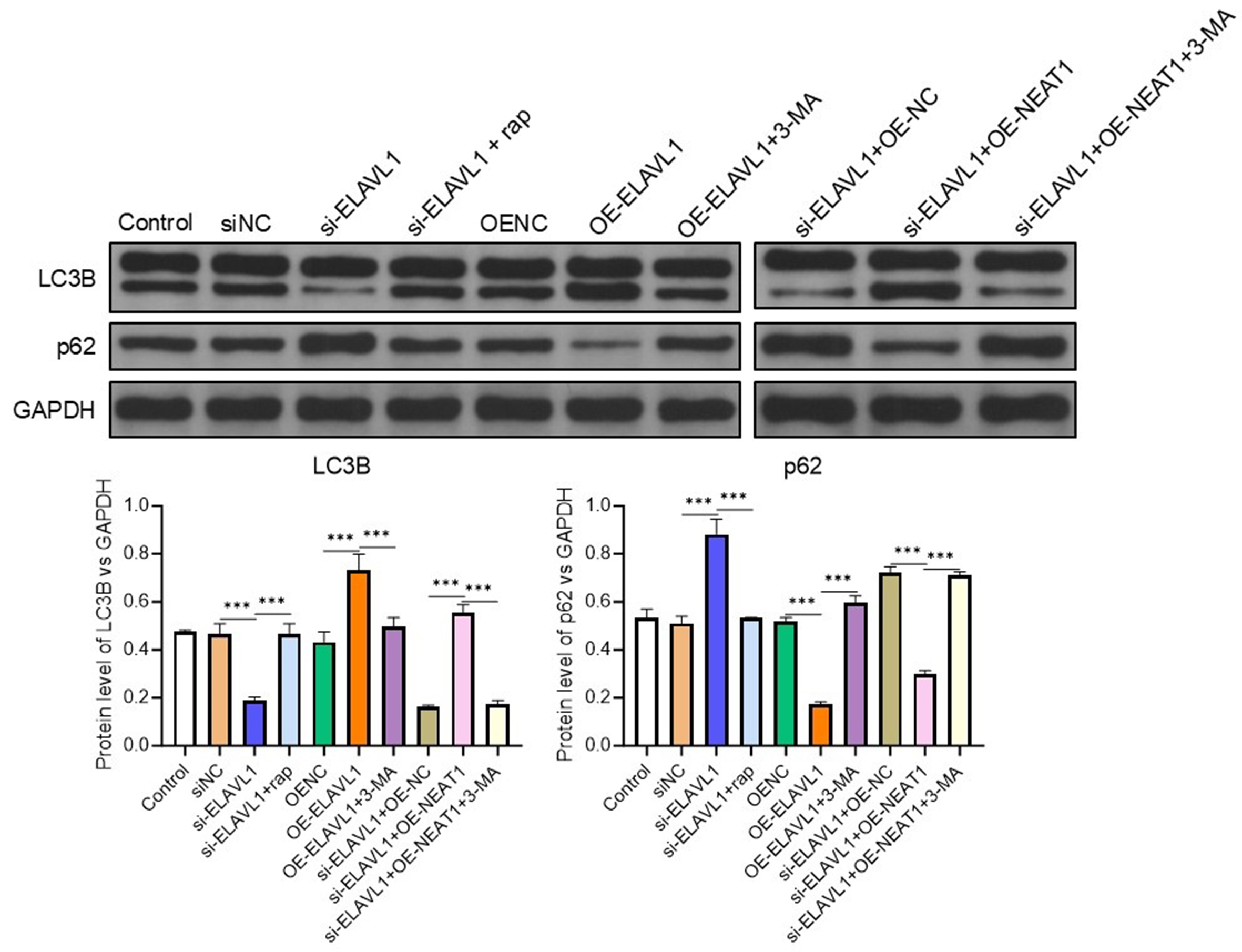

Supplement: FIGURE S1.jpg [file KCBT_A_2469927_SM2684.jpg]
